# Human iPSC-Derived Cerebellar Neurons from a Patient with Ataxia-Telangiectasia Reveal Disrupted Gene Regulatory Networks

**DOI:** 10.3389/fncel.2017.00321

**Published:** 2017-10-13

**Authors:** Sam P. Nayler, Joseph E. Powell, Darya P. Vanichkina, Othmar Korn, Christine A. Wells, Refik Kanjhan, Jian Sun, Ryan J. Taft, Martin F. Lavin, Ernst J. Wolvetang

**Affiliations:** ^1^Australian Institute for Bioengineering and Nanotechnology, University of Queensland, St. Lucia, QLD, Australia; ^2^Queensland Brain Institute, University of Queensland, St. Lucia, QLD, Australia; ^3^Institute for Molecular Bioscience, University of Queensland, St. Lucia, QLD, Australia; ^4^Department of Anatomy and Neuroscience, University of Melbourne, Parkville, VIC, Australia; ^5^School of Biomedical Science, University of Queensland, St. Lucia, QLD, Australia; ^6^Department of Integrated Systems Biology and Department of Pediatrics, School of Medicine and Health Services, George Washington University, Washington, DC, United States; ^7^Illumina, Inc.,, San Diego, CA, United States; ^8^UQ Centre for Clinical Research, University of Queensland, Brisbane, QLD, Australia

**Keywords:** cerebellum, stem cell, differentiation, ataxia-telangiectasia, transcriptome

## Abstract

Ataxia-telangiectasia (A-T) is a rare genetic disorder caused by loss of function of the ataxia-telangiectasia-mutated kinase and is characterized by a predisposition to cancer, pulmonary disease, immune deficiency and progressive degeneration of the cerebellum. As animal models do not faithfully recapitulate the neurological aspects, it remains unclear whether cerebellar degeneration is a neurodevelopmental or neurodegenerative phenotype. To address the necessity for a human model, we first assessed a previously published protocol for the ability to generate cerebellar neuronal cells, finding it gave rise to a population of precursors highly enriched for markers of the early hindbrain such as EN1 and GBX2, and later more mature cerebellar markers including PTF1α, MATH1, HOXB4, ZIC3, PAX6, and TUJ1. RNA sequencing was used to classify differentiated cerebellar neurons generated from integration-free A-T and control induced pluripotent stem cells. Comparison of RNA sequencing data with datasets from the Allen Brain Atlas reveals *in vitro*-derived cerebellar neurons are transcriptionally similar to discrete regions of the human cerebellum, and most closely resemble the cerebellum at 22 weeks post-conception. We show that patient-derived cerebellar neurons exhibit disrupted gene regulatory networks associated with synaptic vesicle dynamics and oxidative stress, offering the first molecular insights into early cerebellar pathogenesis of ataxia-telangiectasia.

## Introduction

The use of patient-specific induced pluripotent stem cells (iPSCs) combined with neuronal differentiation protocols offers great opportunities to gain insight into the genetic basis of neurological disease. A critical factor underpinning the utility of this approach is the ability to reproducibly and robustly differentiate target neuronal cell types and to benchmark their neuroanatomical identity and maturity with their *in vivo* counterparts. The advent of self-inductive/organoid technology, in particular, is starting to bear fruit for investigating complex neurological disorders such as microcephaly (Lancaster et al., 2013) and ZIKA virus-related abnormalities (Dang et al., 2016; Garcez et al., 2016; Qian et al., 2016). To date, these technical developments have not been applied broadly to diseases of the cerebellum, such as ataxia-telangiectasia (A-T). The cerebellum of A-T patients displays loss of Purkinje and granule neurons, resulting in debilitating ataxia (Crawford, 1998). Although several rodent (Kuljis et al., 1997; Borghesani et al., 2000) and pig (Beraldi et al., 2015) models of A-T show evidence of cerebellar abnormalities, none of these models robustly phenocopy the human condition, highlighting the potential value of a human iPSC-derived cerebellar neuronal model (Lavin, 2013).

Protocols for the *in vitro* manufacture of cerebellar neuronal cells aim to recapitulate cerebellar developmental milestones, mimicking *in vivo* development and following a stepwise progression of processes involving neurogenesis, migration, apoptosis, laminar positioning, and synapse formation (Kuhar et al., 1993). After generation of the neural plate and neural tube, the developing embryonic brain organizes into three distinct compartments, namely the prosencephalon (forebrain), mesencephalon (midbrain) and rhombencephalon (hindbrain). The isthmic organizer, a prominent signaling center that secretes inductive cues and growth factors forms a distinct border between the mid and hindbrain territories. During murine development, the isthmic organizer is marked and controlled by the region-specific transcription factors *En1, En2*, *Pax2, Pax5* and *Pax8*, *Otx2* and *Gbx2* (Muguruma et al., 2010). The cerebellum arises from the caudal-most region (the rhombencephalon) of the neural tube. Although cerebellar-like neuronal cells have previously been generated from murine embryonic stem cells (ESCs) (Su et al., 2006; Salero and Hatten, 2007; Muguruma et al., 2010) and human pluripotent stem (PSCs) cells (Muguruma et al., 2015; Wang et al., 2015), it remains unclear whether such *in vitro-*derived cells resemble mature or immature cells of the cerebellum, whether the cells correspond to specific cerebellar lobules/regions or whether they can inform on cerebellar diseases, such as A-T.

Here we show that PSCs can be patterned into primitive cerebellar precursors and that these can be expanded in culture to generate neuronal progeny that strongly resemble early human and mouse cerebellar cell types, as judged by morphological, immunohistochemical and gene expression-based criteria. A novel three-dimensional data visualization tool showing comparison of RNA sequencing data from control and A-T iPSC-derived cerebellar neurons with Allen Brain Atlas datasets reveals gene expression signatures strongly associated with the cerebellum, more so than any other brain region, particularly at 22 weeks post-conception. Analysis of transcriptome changes identifies pathways associated with the formation of the human cerebellum, as expected, but also reveals that absence of ataxia-telangiectasia-mutated (ATM) leads to altered gene regulatory networks involved in neurotransmission and anti-oxidant defense, providing the first insights into the molecular processes underlying cerebellar pathogenesis in a human model for A-T.

## Materials and Methods

### Cell Culture Conditions and Differentiation

Induced pluripotent stem cells were grown and passaged as previously described (Nayler et al., 2012). This study was carried out with approval from and in accordance with the recommendations of The University of Queensland Human Research Ethics Committee (HREC/09/QRCH/103) and performed in accordance with the relevant guidelines and regulations from the Australian Government with written informed consent from all subjects. All subjects gave written informed consent in accordance with the Declaration of Helsinki. Differentiation was performed according to (Erceg et al., 2010). This protocol consists of an initial embryoid-body (EB) formation step, followed by sequential additional of growth factors to floating EB/neurosphere aggregates, plating down on laminin/fibronectin, upon which induction of rosettes/polarized neuroepithelium may be observed. Rosettes harvested for further plating down display cellular outgrowth and expansion, followed by further treatment with growth factors. A diagram in figure two of the results section graphically depicts the protocol. This results in a mixed population of neuralized aggregates and emergent monolayer cells with neuronal-like morphology. Samples exhibited a normal karyotype as assessed by G-band analysis (>15 metaphases analyzed per sample) by a commercial genotyping service. All samples were tested and exhibited negative results using the MycoAlert Mycoplasma detection kit (Lonza).

### Immunostaining

For immunostaining during the differentiation process cells grown on coverslips or chamber-slides were gently washed once with PBS so as not to disturb large spheroid colonies or fragile cell extensions and fixed in 4% paraformaldehyde for 15 min at room temperature. For nuclear staining samples were permeabilized in 0.1% TritonX100/PBS at room temperature for 10 min or ice cold ethanol for 15 min at -20°C, before blocking with 10% goat serum and incubation with the relevant antibodies overnight at 4°C in blocking medium. Antibodies and dilutions used were GBX2 (Abnova) (1:250) and TUJ-1/β-III-TUBULIN (Millipore) (1:1000). Following washing with PBS (3 times 5 min at room temperature) secondary antibodies goat anti-mouse IgG_1_, goat anti-mouse IgG_2B_, goat anti-mouse IgM and Donkey anti-rabbit IgG (Alexa fluor) (2 μg/ml) were used to reveal reactivity. Nuclei were stained with DAPI or Hoechst. This preparation minus the addition of primary antibody was used to confirm specificity of staining.

### Paraffin Embedded Tissue Sectioning and Immunostaining

To more readily allow visualization within the large aggregates that comprised the mixed aggregate/monolayer cultures following 34 days of differentiation, neuronal inductions were pooled and harvested by centrifugation at room temperature (500 g) and fixed in 4% paraformaldehyde at room temperature as a loose aggregate before making into a cell pellet by adding molten agarose, spinning down again then allowing solidification. Once solidified, pellets were processed into paraffin, embedded and sectioned. Sections were mounted on Menzel Superfrost Plus adhesive slides and air-dried overnight at 37°C. Sections were next dewaxed and rehydrated through descending graded alcohols to water before transfer to Tris-buffered saline (TBS) pH 7.6. Endogenous peroxidase activity was blocked by incubating the sections in 2.0% H_2_O_2_ in TBS for 10 min. Sections were washed in three changes of water before transfer of slides to EDTA antigen retrieval buffer pH 8.8 for 15 min at 100°C to heat unmask the antigen from the aldehyde bonds. Slides were allowed to cool for 20 min in the retrieval solution then were washed in three changes of TBS. Non-specific antibody binding was prevented by incubating the sections in Biocare Medical Background Sniper for 15 min.

The following primary antibodies, PAX6 (Developmental studies hybridoma bank) (1:100), ZIC3 (Millipore) (1:100), PTF1α (Abnova) (1:100), and ENGRAILED1 (Millipore) (1:100) were diluted with TBS, applied to the slides and were incubated at room temperature for 60 min. Sections were washed in three changes of TBS before MACH1 Mouse Probe was applied for 15 min at room temperature. Sections were washed in three changes of TBS and MACH1 Universal Polymer HRP was applied for 30 min at room temperature. Sections were washed in three changes of TBS before the signal was developed in betazoid DAB chromogen, MACH1 Kit for 5 min. Sections were then washed in water three times to remove excess chromogen. Sections were lightly counterstained in Haematoxylin, washed in water, dehydrated through ascending graded alcohols, cleared in xylene, and coverslipped. Antibodies were tested against a panel of known controls including tissue from pancreas, kidney, placenta, colon, uterus, lung and melanoma skin, as well as mouse whole brain. Undifferentiated iPSCs or differentiated tissue in the absence of primary antibody showed no positive-immunolabelling, while human tissue panels and mouse cerebella confirmed specificity of staining.

### Flow Cytometry

Hes3-Envy neurons were used for flow cytometry experiments to assess levels of GBX2 expression by harvesting on day 28 of differentiation and processed by washing once in PBS and using either Accutase (Life technologies) to generate a single cell suspension. Cells were fixed in 4% paraformaldehyde for 15 min at room temperature and blocked for 60 min in 10% goat serum before application of primary GBX2 (Abnova) antibody (1:100) overnight at 4°C. Following washing with PBS secondary antibodies goat anti-mouse IgG_1_ were applied. Samples were read on an Accuri C6 flow cytometer and gated based on negative staining controls, prepared identically with the omission of the primary antibody.

### Electrophysiological Recordings and Neurobiotin Electroporation

Following differentiation to stage VI (day 34), cells with neuronal-like morphologies growing freely or out from aggregates as emergent monolayers on coverslips were transferred to normal Ringer solution containing: 130 mM NaCl, 3 mM KCl, 26 mM NaHCO_3_, 1.25 mM NaH_2_PO_4_, 2 mM CaCl_2_, 1 mM MgCl_2_, 10 mM D-glucose and maintained at room temperature prior to electrophysiological recording and single cell dye-electroporation. Electrodes (R ∼4 MΩ) were pulled from borosilicate glass capillaries (Modulohm, Vitrex Medical, Denmark) and were filled with pipette solution containing 2% Neurobiotin^TM^ (Vector Labs, Burlingame, CA, United States) in an artificial intracellular solution containing 135 mM K^+^-methanesulphonate (or KCl), 6 mM KCl, 1 mM EGTA (ethylene glycol bis (2-aminoethyl ether)-N,N,N′,N′-tetraacetic acid), 2 mM MgCl_2_, and 5 mM Na-HEPES (Na–4-2-hydroxyethyl-1-piperazineethanesulfonic acid), 3 mM ATP-Mg^2+^, 0.3 mM GTP-Tris (pH 7.25 with KOH, 305 ± 5 mOsm) (Kanjhan and Vaney, 2008). Under visual guidance (Zeiss Axioskop II), patch electrodes were maneuvered against the soma using a manipulator (MPC-200, Sutter Instrument Company, United States). Once the tip of the electrode touched the cell a negative pressure was applied until a stable and consistent (less than 10% change) membrane seal resistance reading was obtained to record spiking (Kanjhan and Vaney, 2008; Kanjhan and Bellingham, 2013). Seal resistances varied between 20 MΩ and GΩ with a mean value of approximately 220 MΩ. Spikes were recorded from the electrode-cell attached configuration with an Axopatch 1D amplifier (Axon Instruments, Foster City, CA, United States). Signals were acquired at 10 kHz, low pass filtered at 2 kHz, and digitized on a Macintosh computer using Axograph 4.9 software. Several methods were used to facilitate or test spiking of individual cells without spontaneous activity: (1) blowing K^+^ rich (totaling 141 mM K^+^) pipette solution on to the soma of the cell immediately before seal formation; (2) applying slowly depolarizing voltage ramps (from -60 to +10 mV in 4 s); (3) applying depolarizing voltage steps (from -60 to +20 mV at 10 mV intervals for a duration of 0.5–5 s). At the end of recording, spiking cells (i.e., neurons) were individually electroporated with rectangular wave current pulses of ∼400 pA amplitude and 500 ms duration at 1 Hz maintained for 4 min. These electrical pulses were applied via the patch-clamp amplifier to electroporate and dye-fill the individual cells with Neurobiotin (Kanjhan and Vaney, 2008). Measurements described in the results section are listed as mean ± SD.

Upon completion of recording and dye-filling, the cells on the coverslips were fixed in 4% paraformaldehyde in 0.1 M phosphate buffer (pH 7.4) for 15 min, and subsequently washed three times for 30 min in 0.1 M phosphate-buffered saline (PBS). Cells were blocked for 2 h in PBST (PBS with 0.005% Triton-X 100) containing 1% bovine serum albumin (BSA). Cells were then incubated for 2–4 h in Cy3-Streptavidin (1:500 in PBST/1% BSA; Sigma) to visualize dye-fills (Kanjhan and Vaney, 2008; Kanjhan and Bellingham, 2013). Cells were washed in 0.1 M PBS and mounted with a standard glycerol-based mounting medium. Cells were imaged with an Olympus BX61 (Olympus Fluoview ver. 1.7c) confocal microscope using imaging software Olympus Fluoview ver. 1.7c.

### RNA Isolation, cDNA Synthesis, and Q-PCR

Total RNA was extracted using *RNA Spin II* isolation columns (*Macherey Nagel*). On column digestion of DNA with RNase free *DNase* was performed according to the manufacturer’s specifications (*Ambion*, Austin, TX, United States). Concentration and 260/280 ratios were quantified using a NanoDrop 1000 spectrophotometer before synthesis of cDNA using iScript cDNA synthesis kit (Bio-Rad) according to the manufacturer’s specifications (500 ng). Primers were designed to span exon–exon boundaries to exclude the possibility of genomic DNA contamination contributing to PCR signal, as well as DNase treatment of RNA. Primer efficiencies were calculated using pooled cDNA libraries. Melt curves indicated primers were specific via the presence of a single sharp peak. A minus *Reverse transcriptase* and no template controls were included to control for reaction contamination/specificity. E16 BL6 mouse cerebellum (a kind gift from Dr Conor O’Leary, The University of Queensland) was used as a positive control. *ACTB* was chosen given its steady expression at relevant time points. Relative expression data was calculated using the Δ*C*t method. Primers were *ACTB*-F (*GCCGGGACCTGACTGACTAC*), *ACTB*-R (*TTCTCCTTAATGTCACGCACGAT*), *OTX2*-F (*CACTTCGGGTATGGACTTGC*), *OTX2*-R (*GTGAACGTCGTCCTCTCCC*), *GBX2*-F (*AAAGAGGGCTCGCTGCTC*), *GBX2*-R (*GGTCGTCTTCCACCTTTGAC*), PAX6-F (AGCTAGCTCACAGCGGGG) PAX6-R (TCTGATGGAGCCAGTCTCGT), EN1-F (TCAAAACTGACTCGCAGCA), EN1-R (TCAAAACTGACTCGCAGCA), MATH1-F (AACAGGTGAATGGGGTGCAG), MATH1-R (CTCGGACAAGGCGTTGATGT), HOXB4-F (GCAAAGAGCCCGTCGTCTAC), and HOXB4-R (CGTGTCAGGTAGCGGTTGTA).

### RNA Sequencing Library Preparation and Analysis

After 34 days of differentiation (Erceg et al., 2010), RNA was harvested from control (C11) and A-T (AT30, a compound heterozygote 8368delA and 7570delG) neuronal inductions, as well as from their undifferentiated iPSC counterparts (sorted by flow cytometry for pluripotency surface marker TRA-1-60). At time of harvest, day 34 samples were grown in terminal/stage-VI media, containing BMP4, BMP6, BMP7, GDF7, SHH, BDNF, JAG1, and NT3. All samples were harvested by collecting RNA from three individually cultured/differentiated wells. RNA was isolated as described in above section. All RNA samples were subject to RNA integrity analysis using the RNA 6000 Nano total RNA kit (*Agilent*), and recorded a RIN (RNA integrity number) in excess of 8.5. One μg of RNA from each replicate sample was used to generate a library using the TruSeq Stranded Total RNA Sample kit (*Illumina)* as per the manufacturer’s specifications. Libraries were generated and sequenced across two lanes of a HighSeq 2500, using the rapid run protocol to obtain 100 bp paired-end reads.

### RNA Sequencing Data Processing

After read quality control using FastQC (Andrews, 2012), primary alignment to the reference human genome (hg19) was carried out using the Subread (Liao et al., 2013) version 1.18 package for R version 3.2.1 with default mapping parameters. Reads were summarized and annotated to Ensembl version 69 transcripts and genes using the feature Counts function of Subread version 1.18. Details can be found at website^1^, section 1.6.

### Differential Gene Expression Analysis, Gene Ontology Analysis and Pathway Analysis

Differential expression analysis was conducted using the R package for statistical computing version 3.2.2 using the limma (Ritchie et al., 2015) library version 3.26.8. Genes were tested for differential expression if they displayed one count per million in all replicates of at least one phenotypic group. We used limma’s Voom function to modify linear model fitting parameters based on the mean variance trend of counts observed. Input counts were TMM (Trimmed mean of *M*-values) (Robinson and Oshlack, 2010; Robinson et al., 2010) normalized and median-centered in addition to the log2 and count-per-million transforms applied by Voom. For analyses, genes were considered differentially expressed with a Benjamini–Hochberg adjusted *p*-value < 0.01 and a fold change of 1.5 or greater.

Three differential expression comparisons were generated; (1) day 0 vs. day 34 of differentiation for AT30, (2) day 0 vs. day 34 of differentiation for C11, to explore respective differentiation outcomes and (3) AT30 day 34 vs. C11 day 34, to explore disease-specific changes following cerebellar differentiation (full tables are shown in **Supplementary Tables S2**–**S4**, respectively). Volcano plots were used to graphically illustrate the differential expression of genes, by plotting the log fold-change (LFC) against the FDR (false discovery rate, a measure of robustness).

Processed data, RPKM (Reads Per Kilobase of transcripts per Million mapped reads) and TMM normalized, log2 transformed data is available in Stemformatics^2^ (Wells et al., 2013). Raw data and count tables have been deposited to the Gene Expression Omnibus (GEO) under accession GSE75852.

Gene ontology analysis was carried out using the g:Profiler (g:Cocoa) (Reimand et al., 2011) package using a cutoff of 1.5logFC. Pathway analysis was performed using *Advaita iPathway Guide*^3^ using a logFC cutoff of 1.5 and adjusted *p*-value of 0.01.

### Cross-Study Analysis Using Allen Brain Atlas Datasets

We performed a cross-study analysis using transcriptional data from the Allen Brain Atlas to verify the expression profile of the differentiated cells. Analyses were performed to determine if the expression profile of the control (C11) and A-T (AT30) differentiated cerebellar neurons matched those of cells located in the cerebellum and to identify the developmental time points they were most similar to.

We downloaded microarray expression data collected from six postmortem adult individuals from The Allen Brain Atlas Human Brain Map^4^ and developing brain data from four samples, two at 15–16 weeks and two at 21–22 weeks. For each sample, expression data measured in tissue from 364 to 947 unique micro-dissected regions of the adult brain. We filtered the ABA expression data to contain only transcripts and regions that were expressed or assayed in two or more samples, respectively. After filtering, 501 unique regions and 13,829 transcript probes remained. The 501 regions can be assigned to three anatomical structures in the brain: the brain stem (BS, *n* = 59), the cortex (CX, *n* = 362), and the cerebellum (CB, *n* = 80).

For each of the transcripts, normalized expression levels were averaged and ranked for each region separately for adult, 15–16 weeks and 21–22 weeks samples. We also averaged and ranked the expression levels for transcripts expressed in the C11 and AT30 differentiated cells. For both C11 and AT30 we assigned a rank difference value (Brennand et al., 2015) for each transcript per region, again separately for adult, 15–16 weeks and 21–22 weeks samples. We then performed a Wilcoxon’s rank-sum test to assess the strength of the matched rank between each of the 501 ABA regions, across adult and developmental times points and the C11/AT30 differentiated cells. Three-dimensional reconstructions were made using the plotly package^5^ for R. Nomenclature for naming brain regions is consistent with that used by Allen Brain Atlas as described by (Schmahmann et al., 1999).

### Statistical Methods

Statistical methods have been outlined/referenced in their respective sections where appropriate. qPCR data was tested for significance by utilizing an unpaired *t*-test (Mann–Whitney), significance denoted by *p*-values < 0.05. Statistical methods employed by the gProfiler/gCocoa suites are detailed in (Reimand et al., 2011). Statistics pertaining to sequencing libraries, mapping, normalization and differential-expression analyses have been referenced and/or are included at website^6^, section 5. A Wilcoxon’s rank-sum test was used to assess the strength of the matched rank between Allen Brain Atlas samples and iPSC-derived differentiation experiments. *Advaita iPathway Guide* statistical details can be viewed (Draghici et al., 2007).

## Results

### Generation of Cerebellar-Like Neuronal Cells

**FIGURE 1:**
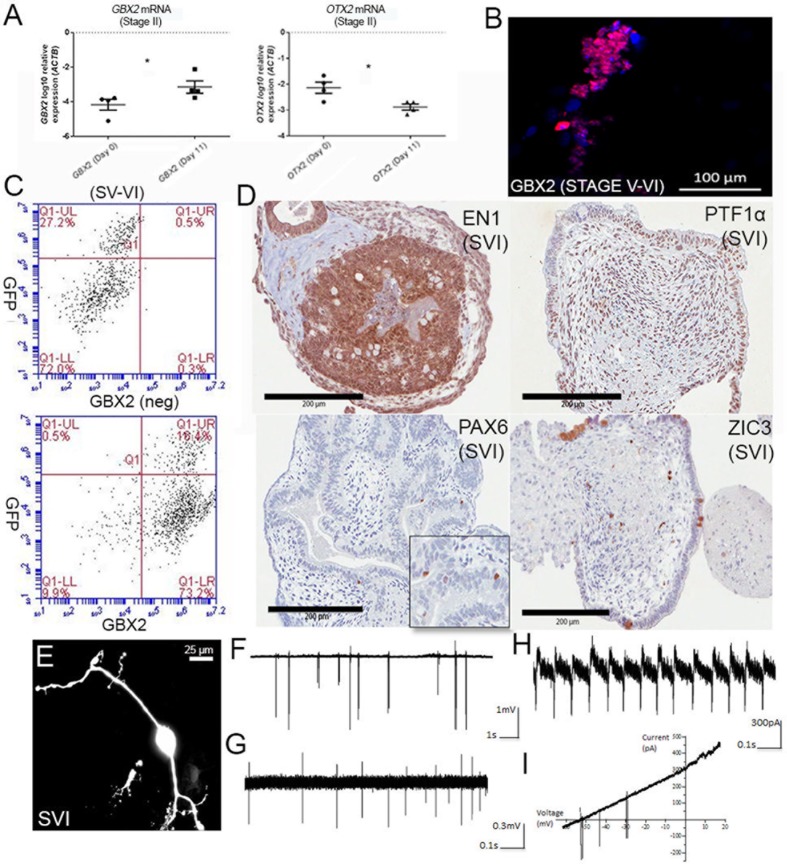
Differentiation of cerebellar neurons. **(A)** Upregulation of *GBX2* mRNA and downregulation of *OTX2* mRNA after 11 days of differentiation. Samples were normalized to *ACTB* and show SEM from four independent differentiation experiments (2 iPSC, 2 hESC). ^∗^Denotes statistical significance, *p*-values =< 0.05, unpaired *t*-test, Mann–Whitney. **(B)** Immunofluorescent labeling for GBX2 following 28 days of differentiation. Nuclei counter-stained with DAPI (scale bars are 100 μm). **(C)** Flow cytometry for GBX2. Top panel shows negative control. Lower panel shows GBX2 immunolabelling in Hes3-Envy following 28 days of differentiation (*x*-axis). **(D)** Serial sectioning and H&E staining of paraffin-embedded cell pellets following 34 days of differentiation displays reactivity with antibodies directed against hindbrain markers EN1, PTF1α, PAX6, and ZIC3 (scale bars are 200 μm). Inset is shown for PAX6. **(E)** Morphology of representative neuron following dye-filling with Neurobiotin electroporation (scale bars are 25 μm). **(F–I)** Electrophysiological recordings.

Given no subsequent reports have detailed the use of the protocol from Erceg et al. (2010) we first verified that this method reliably gave rise to cells with a hindbrain-like identity. Human ESCs H9 and Hes3/Hes3-Envy (Thomson et al., 1998; Reubinoff et al., 2000; Costa et al., 2005) provided by the Australian Stem Cell Centre, as well as human iPSC lines previously generated in our laboratory were differentiated independently, according to the protocol. **Figure 1A** shows induction of the hindbrain-specific transcription factor *GBX2* following the first two stages of differentiation (11 days in culture) and concomitant downregulation of *OTX2* in all four wild-type differentiation experiments. Between stages V and VI (day 28), immunolabeling confirmed nuclear GBX2 expression in the majority of cells (**Figure 1B** shows a representative image), suggesting near homogeneous transition into a hindbrain fate. Flow cytometry confirmed that approximately 90% of the population expressed GBX2 protein (**Figure 1C**).

A degree of self-organization reminiscent of the developing cerebellum was observed in Hematoxylin and Eosin (H&E) stained serial sections, following 34 days of culture. We observed foliation and a thickened layer at the periphery of the aggregate borders, reminiscent of the developing external granule layer (EGL) in the cerebellar cortex, in a subset of aggregates (**Figure 1D**). Others showed polarized neuroepithelium, consistent with reports from (Muguruma et al., 2015). Immunolabeling with antibodies directed against the hindbrain markers ENGRAILED1, PTF1α, or PAX6 and ZIC3, markers of developing granule neurons, indicates a profile consistent with enrichment for cerebellar progenitors and early granule cell differentiation. Interestingly, the core of the aggregates showed widespread expression of early precursor markers (ENGRAILED1 and PTF1α), while a tendency toward ZIC3 expression was noted toward the periphery of the spheres.

Further culture of cell aggregates followed by disaggregation and plating permitted the outgrowth of neuronal cells that exhibit morphology consistent with cerebellar neurons at distinct stages of development (Supplementary Figure S1). This included neurons with morphologies consistent with those of granule neurons (Supplementary Figure S1A) featuring a small soma (<10 μm), T-shaped branching axons (Supplementary Figure S2) and the characteristic short, stubby claw-shaped appendages first described by Ramon Cajal (De Carlos and Borrell, 2007). Also noted were several other distinct morphologies, including cells displaying a large pear-shaped soma (>20 μm), and bifurcated branching (Supplementary Figure S1B). We conclude that delivery of factors involved in early isthmo-cerebellar development reported by Erceg et al. (2010) was sufficient to instigate hindbrain-specific patterning of human PSCs.

### Cerebellar Neurons Show Signs of Electrophysiological Maturity

Morphological analysis of dye-filled/fluorescently labeled neurons growing as monolayers or emerging from plated aggregates (**Figure 1E**) revealed the presence of bipolar and multipolar distributions of cellular processes, indicative of polarization of axonal and dendritic components also observed in early cerebellar neurons. Dendrites displayed up to 4th order branching and a reduction in their diameter as they further branched close to the soma. Electrophysiological recordings from a total of 11 neuronal cells revealed spontaneous or evoked spiking activity upon sealing of the somatic membrane to the tip of the electrode (**Figures 1F–I**). A subset of neurons (*n* = 5) only fired upon depolarization brought about by blowing high K+ (141 mM) from the pipette solution immediately prior to seal formation, or by application of slow voltage ramps (-60 to +20 mV at 4 s) once a stable seal formed (**Figure 1I**). Cells with spiking activity were considered neurons, whereas cells without spiking activity were likely either glial or immature cells (not included in the current study). The firing frequencies of individual neurons ranged from 0.1 to 20 Hz (5.4 ± 7.2 Hz). Such large variation in spiking frequency most likely reflects varying developmental stages or maturity of individual cells, or may indicate presence of multiple neuronal subpopulations. The spiking patterns were usually irregular (10 out of 11 cells; **Figures 1F,G**) although one cell displayed regular spiking (**Figure 1H**). Heterogeneous spiking activity likely indicates developing intrinsic properties of fetal cerebellar neurons, with limited or no synaptic inputs.

### Transcriptomic Profiling of iPSC-Derived Control and A-T Cerebellar Neurons Following Differentiation

**FIGURE 2:**
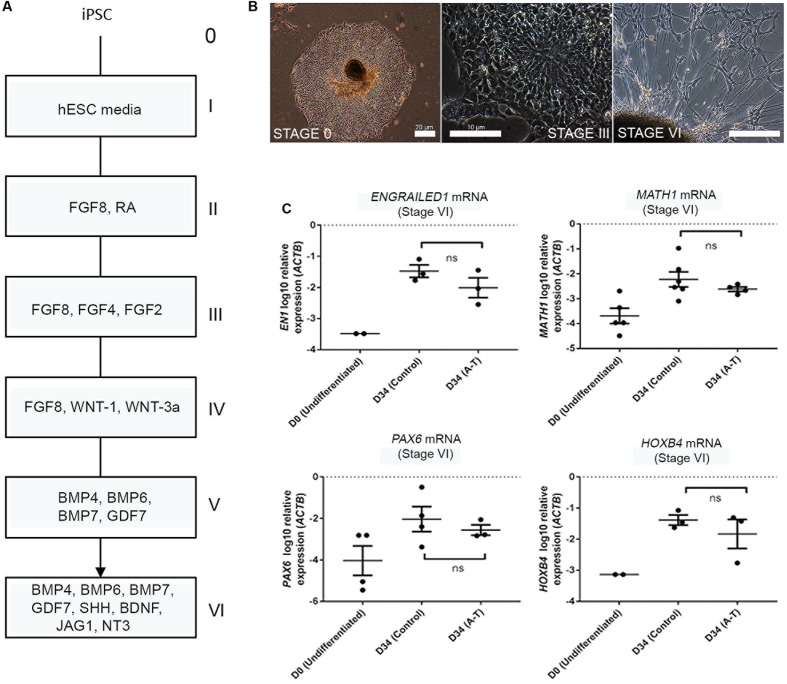
Cerebellar differentiation of control and A-T iPSCs **(A)** schematic detailing differentiation protocol. Individual stages are denoted by roman numerals; boxes show relevant growth factors/recombinant proteins used at each stage. **(B)** Representative phase-contrast photographs of stage I (undifferentiated iPSCs), stage III (polarized neuroepithelia/rosettes) and stage VI (final plating stage, showing mixed aggregate/monolayer culture). **(C)** Upregulation of *EN1, MATH1, PAX6* and *HOXB4* mRNA is comparable between A-T and control iPSCs following 34 days of differentiation (stage VI). Samples were normalized to *ACTB* and show SEM from independent differentiation experiments comprising between 3 to 6 control/A-T differentiations. Values for neural-specific markers in which day 0 samples did not generate *C*t-values have not been included. *p*-values = *EN1* (0.4, ns), *MATH1* (0.3524, ns), *PAX6* (0.6286, ns), *HOXB4* (>0.999, ns), unpaired *t*-test, Mann–Whitney.

Given that rodent models of A-T do not accurately recapitulate the cerebellar ataxia of patients with A-T, it has remained unclear whether A-T is a neurodevelopmental defect, a degenerative process, or both. Having established that the differentiation protocol gives rise to a mixed population of developmentally immature neuronal types, we transcriptionally profiled these cells to identify the subtypes present, benchmarked their maturity and compared control and A-T patient transcriptomes to identify disease-specific features. To test the feasibility of this approach we first compared mRNA levels of key cerebellar genes, following differentiation of two control iPSCs (FB and C11) and two A-T patient iPSCs (AT30 and AT34) generated in our lab (Nayler et al., 2012; Briggs et al., 2013). Full details of all cell lines utilized are outlined in Supplementary Figure S3. Cells were differentiated according to (Erceg et al., 2010), alongside hESC lines H9, Hes3 and Hes3-Envy (Costa et al., 2005). **Figure 2A** shows a diagrammatic outline of the differentiation time-course, with roman numerals denoting key stages. Representative images are shown in **Figure 2B** to illustrate stages at which cells were sampled (i.e., stage I/day 0 and following stage VI (final plating before harvest on day 34).

Q-RT PCR profiling of cells following 34 days of differentiation revealed the expected induction of hindbrain/cerebellar markers *EN1, MATH1, PAX6*, and *HOXB4* (**Figure 2C**) and importantly no statistically significant differences in expression levels of these markers between control and A-T samples (*p*-values 0.4, 0.3524, 0.6286 and >0.999, respectively). We interpreted these data to indicate that ATM is unlikely to interfere with gross cerebellar differentiation itself and therefore may allow us to identify gene expression changes resulting from absence of *ATM*, rather than cellular make-up.

### A-T and Control iPSC-Derived Cerebellar Neurons Share Highly Similar Post-Differentiation Transcriptomes, Resembling the Developing Human Cerebellum

**FIGURE 3:**
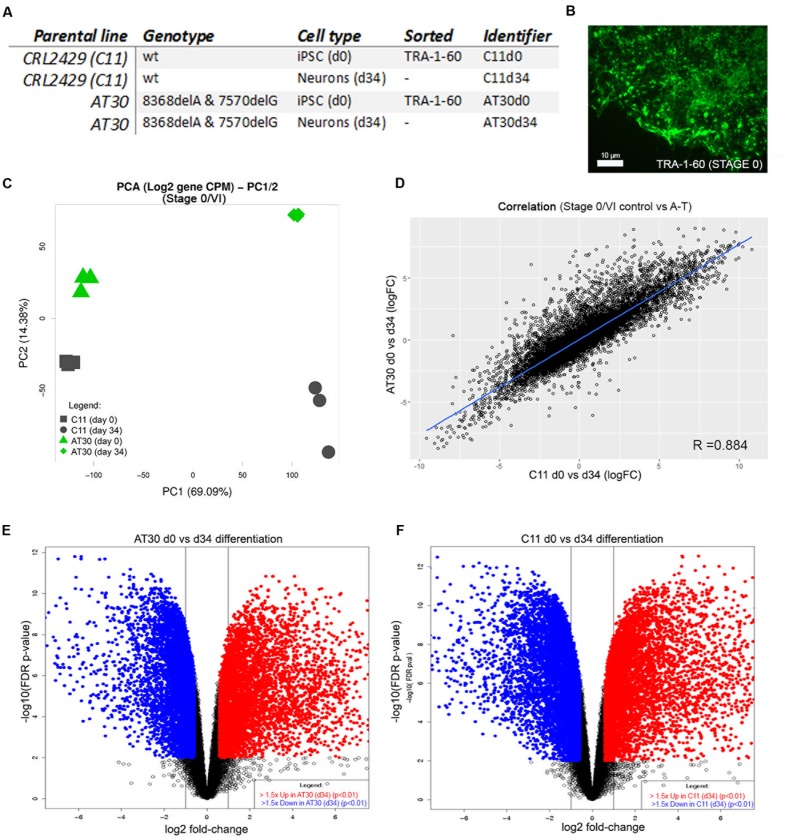
Transcriptomic characterization of differentiated neurons. **(A)** Table detailing iPSCs selected for RNA sequencing. **(B)** Representative iPSC colony immunolabelled with antibody to TRA-1-60. Scale bar is 10 μm. **(C)** PCA shows tight clustering of experimental replicates. *Y*-axis shows PC2, *X*-axis shows PC1. **(D)** Correlation between samples following differentiation by limma-estimated log-fold change in expression between AT30 d0 vs. d34 and C11 d0 vs. d34. **(E,F)** Volcano plots show changes in the expression of genes after differentiation (**E** = AT30 d0 vs. d34, **F** = C11 d0 vs. d34). Differentially expressed genes (adj. *p*-value < 0.01 and logFC > 1.5) are shown as blue/red points.

We next isolated RNA from each of three differentiation experiments from one control line (C11) and one ATM mutant (AT30) on day 34 of the protocol. RNA was also harvested from each of three C11 and AT30 iPSC cultures following surface labeling of live cells for TRA-1-60 and retrieval by flow cytometry on Day 0/Stage I of the differentiation procedure; Details of iPSCs used are shown in **Figure 3A**. **Figure 3B** shows a representative adherent iPSC colony following labeling for TRA-1-60. Samples were treated in accordance to proper RNA-handling techniques and subjected to RNA sequencing using the Illumina TruSeq Stranded Total RNA Sample workflow. Read depth and mapping statistics are shown in **Supplementary Table S1**. Principal component analysis (**Figure 3C**) shows tight clustering of experimental replicates which separate majorly following differentiation (PC1, 14.38%, *Y*-axis and PC2 69.09%, *X*-axis).

A-T and control neurons shared highly similar transcriptomes following differentiation, as indicated by a high degree of correlation (*R* = 0.884) between differentially expressed genes (**Figure 3D**). This was further supported by shared similarity in gene ontology enrichment (Supplementary Figure S4 and **Table S5**). Strong enrichment was observed for genes associated with *brain development* (adj. *p*-values = 5.33E-12/1.35E-04, AT30/C11, respectively), *anterior/posterior pattern specification* (7.83E-10/2.18E-12), *nervous system development* (3.28E-43/2.27E-21), *regulation of neuron differentiation* (1.27E-14/1.08E-06), *generation of neurons* (3.61E-26/5.92E-14), *gliogenesis* (1.45E-07/1.87E-05), *positive regulation of neuron differentiation* (2.11E-10/6.08E-04), *regulation of nervous system development* (3.54E-18/2.45E-10), *regulation of stem cell differentiation* (3.85E-02/6.17E-06), *neurogenesis* (4.60E-30/4.35E-17) and *neuron development* (7.00E-17/1.22E-08). Full reports including pathway representation and individual/overlapping genes involved are included in **Supplementary Table S5**.

Volcano plots depicting differential gene expression analysis from AT30 (**Figure 3E**) and C11 control (**Figure 3F**) illustrate genes changing following differentiation (log2 fold change, *x*-axis) and statistical significance (-log10 FDR/*p*-value). Pluripotency-associated genes including *NANOG, SOX2*, and *OCT4* were downregulated, as expected. Analysis confirmed the upregulation of genes with known roles in establishment of the cerebellar primordium: *EN1* (5.37/1.79 logFC in AT34/C11, respectively), *HOXB4* (4.62/4.45 logFC), *HOXA2* (4.49/3.31 logFC), or granule cell generation; *MEIS1* (6.80/4.97 logFC), *MEIS2* (4.96/2.98 logFC), *GAS7* (2.32/2.9 logFC), *NFIC* (2.62/1.78 logFC), and *SHH* (5.49/5.74 logFC).

The expression of *PAX6* (2.06/2.46 logFC) and *UNC5-C/NETRIN-1* (logFC 5.85/3.78) was consistent with granule cell migration. A number of genes involved with Purkinje cell development, maintenance and migration were detected including *NGF* (3.56/3.52 logFC), *RELN* (5.08/5.49 logFC), and *BDNF* (2.55/2.56 logFC). Glial cell markers *ERBB4* (4.06/3.90 logFC), *GJB6* (6.24/7.01 logFC) and *WIF1* (1.08/3.73 logFC) were also detected, as well as markers of astrocytic-dependent developmental synaptogenesis *SPARCL1* (5.03/5.38), *THBS1* (2.02/1.85), *THBS2* (3.20/2.02) and *SPARC* (5.23/4.09).

### Allen Brain Atlas-Based Analysis Shows iPSC-Derived Neurons from A-T and Control Donors Are Transcriptionally Similar to the Transcriptome of the 22-Week Fetal Cerebellum

**FIGURE 4:**
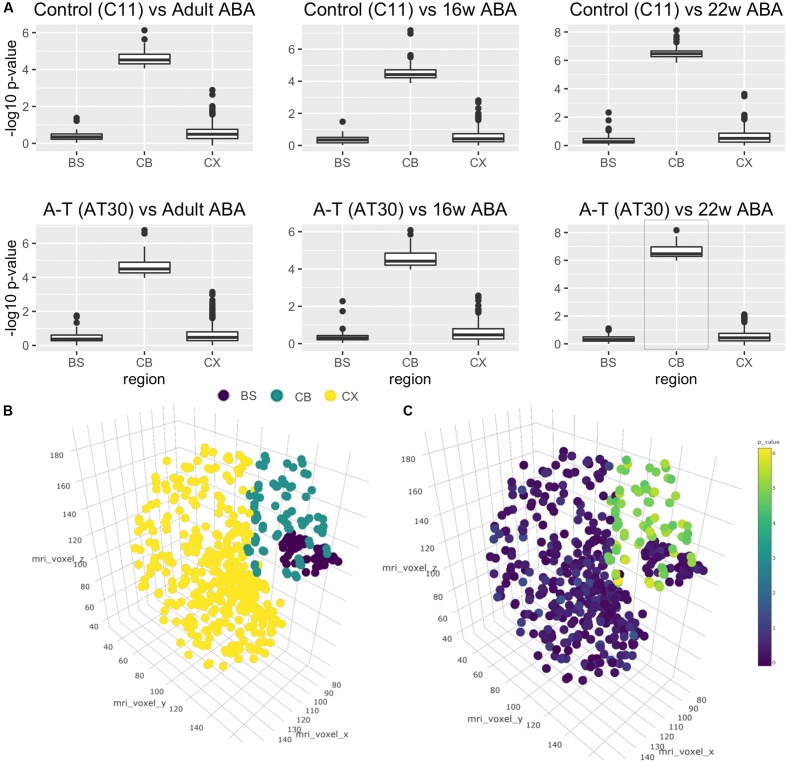
Cross-study analysis using transcriptional data from the Allen Brain Atlas. **(A)** Wilcoxon’s rank-sum tests shows transcriptional similarity between brain regions and C11 (top)/AT30 (lower) cerebellar neurons. **(B)** Three-dimensional reconstruction of Allen Brain Atlas data demonstrating discrete regions (BS, brain stem; purple/CB, cerebellum; teal/CX, cortex; yellow). **(C)** Three-dimensional reconstruction of Allen Brain Atlas samples showing -log10 *p*-values pertaining to comparison of differentiated cells to Allen Brain Atlas data from that region.

A major issue for the field of disease modeling with iPSC-derived neurons is to benchmark their developmental age, in most cases reported to be at embryonic-stage maturity. Using the Allen Brain Atlas, a landmark resource detailing stage-specific regional profiles of the human brain, as an independent comparator, and methods previously used to assess cortical neuronal identity (Kang et al., 2011; Stein et al., 2014; van de Leemput et al., 2014) we next compared the transcriptomes of control and A-T iPSC-derived cerebellar neurons to qualify the region-specific neuronal identity and to approximate their stage of developmental maturity. Our analyses revealed that cerebellar neuronal transcriptomes were highly similar to the Allen Brain Atlas cerebellum (CB) samples, at each of the developmental time points included in our comparison (adult, 16 and 22 weeks post-conception), and significantly more similar to the CB than the brain-stem (BS) or the cortex (CX). Wilcoxon’s rank-sum test -log10 *p*-values (**Figure 4A**) when compared to Adult Brain in C11 and AT30, respectively, were 0.39/0.47 (BS), 0.58/0.60 (CX) and 4.60/4.64 (CB) and relative to 16 weeks post-conception, *p*-values were 0.36/0.37 (BS), 0.54/0.59 (CX), and 4.55/4.53 (CB). The strongest statistical similarity (**Figure 4A**, lower right panel, highlighted) was with that of the 22-week old fetal cerebellum 6.51/6.64 (CB), 0.41/0.36 (BS), and 0.62/0.54 (CX). As expected, undifferentiated iPSCs exhibited -log10 *p*-value rank-sum scores ranging between 0 and 2, significantly less than observed for the differentiated cells. This provides a robust, independent molecular validation of the *in vitro*-derived neurons as a legitimate model of cerebellar development.

To better visualize these data, we performed three-dimensional brain reconstructions using voxel coordinates pertaining to discrete regions from the Allen Brain Atlas (**Figure 4B**). Reconstruction displays separated and colored groups by region (BS = Brain stem, purple/CB = Cerebellum, teal/CX = Cortex, yellow). **Figure 4C** shows a three-dimensional brain reconstruction for the strongest correlation from **Figure 4A** (AT30 vs. 22 weeks post-conception, highlighted in **Figure 4A**) where -log10 *p*-values are projected as colored dots in three-dimensional space according to the statistical comparison with the region at that coordinate (statistical analyses are available in **Supplementary Table S6**, remaining three-dimensional reconstructions are shown in Supplementary Figure S5). An interactive, online three-dimensional plot is available at website^7^.

Further detail is shown in Supplementary Figures S6–S8, which show flattened heatmaps displaying *p*-values pertaining to each fine brain region. Interestingly, the three most statistically significant areas in this comparison were lobule III (Centralis, anterior lobe, -log10 *p*-value = 8.17), lobule X (Nodulus, vestibulocerebellum, -log10 *p*-value = 7.73) and lobule VIIIA (Pyramus, posterior lobe, -log10 *p*-value = 7.63). Collectively, these data demonstrate that iPSC subjected to *in vitro* differentiation with this protocol are transcriptionally more similar to 22-week old fetal human cerebellum than any other brain region, and show characteristic signatures resembling particular anatomical regions of the cerebellum.

### *ATM* Transcript Is Downregulated during *in Vitro* Cerebellar Differentiation

We next examined levels of *ATM* transcript in control and A–T samples over the cerebellar induction time course.

This is particularly pertinent as the mutations carried by AT30, used in the current study, are predicted to result in mRNA instability via non-sense-mediated decay. Interestingly, our data reveal *ATM* is downregulated during differentiation in both the control and the A-T cerebellar induction (Supplementary Figure S9).

AT30 is a compound heterozygote, with two deletions in the *ATM* cDNA (8368delA and 7570delG; hg19 genomic coordinates chr11:108214045 and chr11:108202224), which introduces a TGA stop codon at cDNA positions 7591–7593 and 8413–8415 (hg19 genomic coordinates chr11:108,202,247–108,202,249 and chr11:108,214,094–108,214,096, respectively). Indeed, we detected fewer reads mapping to the *ATM* locus in both undifferentiated AT30 iPSCs and after 34 days of cerebellar differentiation. Both mutations were confirmed in the RNAseq data using IGV (Supplementary Figures S10, S11).

### Differential Expression and Pathway Analysis of Control and A-T Cerebellar Neurons Reveals Synaptic Vesicle, Metabolic and Oxidative Stress Defects in A-T

**FIGURE 5:**
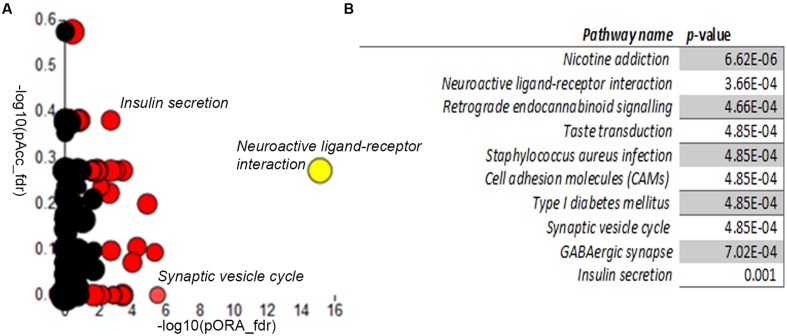
Differential gene expression pathway analysis. *Adavita iPathway Guide* analysis shows statistically–significantly disrupted pathways **(A)** Perturbation (*y*-axis) vs. Overrepresentation (*x*-axis) reveals disrupted pathways further shown in **(B)** Table showing statistically–significantly altered pathways in AT30, compared with C11 cerebellar neurons.

We next compared the control and A-T cerebellar neuron transcriptomes using *Advaita iPathway Guide* analysis. Gene-set enrichment analysis utilizing combined measures of pathway over-representation and transcript perturbation levels allowed us to identify pathways featuring statistically significant disruptions. *Neuroactive ligand-receptor interaction* (*p-*value 3.66E-04), *Type I diabetes mellitus* (4.85E-04), *Synaptic vesicle cycle* (4.85E-04), *GABAergic synapse* (7.02E-04) and *Insulin secretion* (0.001) pathways were among those identified as significantly altered in day 34 A-T cerebellar neuronal cultures, relative to controls (**Figures 5A,B**). Gene membership of select pathways (highlighted in **Figure 5A**) and expression levels are outlined in Supplementary Figures S12–S14. **Supplementary Table S4** provides the full list of genes that are differentially expressed between the two genotypes.

## Discussion

The cerebellar degeneration associated with A-T has remained poorly understood, in part due to the lack of a suitable animal model. Here we applied a protocol for the generation of cerebellar neurons from human control and A-T iPSCs to gain insight into early cerebellar development and potential pathogenic mechanisms brought about by ATM deficiency. We validated an existing cerebellar differentiation protocol (Erceg et al., 2010) in several PSC lines and increased the level of characterization performed previously by using RNA sequencing. To facilitate access to our dataset we have made available raw and processed data in GEO and also Stemformatics (Wells et al., 2013) an open-access, browser-based stem cell transcriptomics resource.

Over the course of the differentiation, we detected early upregulation of *GBX2*, a transcription factor that serves to define the mid-hindbrain barrier. This process is consistent with the *in vivo* downregulation of *OTX2* by *GBX2*, which takes place in the first rhombomere, and is a definitive step in cerebellar development from the mid-hindbrain junction (Sato et al., 2004). Differentiating cells downregulated pluripotency factors and transited through a stage where the majority expressed GBX2, indicating near-uniform acquisition of a hindbrain neuronal fate. The resulting neuronal cells expressed key cerebellar markers, exhibiting polarized neuroepithelium reminiscent of the ventricular zone, and also later a thickened outer layer and foliation, reminiscent of the cerebellar EGL. Interestingly, our data suggest that the protocol of Erceg et al. (2010) consists of a stage where embryoid-bodies undergo a degree of self-organization similar to that exhibited by Muguruma et al. (2015).

The expression of several markers found in distinct cell types and stages of cerebellar development showed strong parallels between the *in vitro* differentiated model and human fetal cerebellar development. These markers included ENGRAILED1, which plays a pivotal role in controlling segmentation in the dorsal midbrain and anterior hindbrain. In the mouse *Engrailed1* exhibits sustained expression within the cerebellum up until P21 (Wilson et al., 2011). Our iPSC-derived cerebellar neurons also showed strong upregulation of *SHH*. Sonic hedgehog signaling assists development of the cerebellum through several modes of action – it is required by granule cell progenitors and Bergmann glia for correct proliferation and differentiation (Dahmane and Ruiz i Altaba, 1999) and is also secreted by Purkinje cells to guide the migration of granule cells to their correct position within the IGL (internal granule layer) (Grimmer and Weiss, 2008).

In a recent review on neural-organoid cultures, Kelava and Lancaster (2016) highlight the trade-off between homogeneity and complexity, distilling the challenge of using facile homogenous cellular models as opposed to mixed cultures which resemble the natural configuration of the brain. Our RT-PCR, immunohistochemical and electrophysiological data substantiate a degree of cellular heterogeneity present during differentiation, a major hurdle for the field to address with respect to neural differentiation and disease modeling. We cannot exclude that some variability in gene expression may have arisen due to differential presence of specific neuronal cell types at various stages of developmental maturity. For example in control cells both *BMP6* and *NBL1* were expressed but not *BMP7*, suggesting a recent departure from an *MATH1*-expressing proliferative granule cell population (Krizhanovsky and Ben-Arie, 2006; Grimmer and Weiss, 2008). A-T cerebellar neurons on the other hand still expressed *MATH1* suggesting a slower commitment of rhombic lip progenitors to fully differentiated granule neurons.

Our transcriptome analyses of cerebellar neurons generated over 34 days makes use of unbiased, higher order bioinformatics classification strategies which indicate that the cellular composition was largely comparable between control and A-T mutants. Three-dimensional mapping of ranked-gene expression data demonstrates statistically significant spatial similarity to micro-dissected regions of the human cerebellum. Temporally and spatially both control and A-T neurons at day 34 of culture are transcriptionally most similar to the 22-week post-conception human cerebellum. In this respect iPSC-derived cerebellar cells resulting from this protocol may undergo more rapid maturation *in vitro* to other reported iPSC-derived cerebral neuronal cell types. Importantly, transcriptome comparison of our iPSC-derived cerebellar neurons with datasets from the Allen Brain Atlas revealed an overwhelming similarity to lobule III, VIIIa and other lobules of the cerebellum. These structures have been linked with coordination of hand-eye movement and oculomotor deficits that are early clinical phenotypes observed in A-T patients.

Differential gene expression analysis of control and A-T cerebellar neurons supports several cellular and animal model-derived theories that seek to explain the nature of the neurodegeneration in A–T. These include the commonly held theory (Wang et al., 2011; D’Souza et al., 2012) that ROS levels are misregulated in A-T and adversely affect the cerebellum. Our data show that two of the most highly upregulated genes in A-T neuronal cells were *GSTT1* (8.20 logFC in A-T relative to controls) and *CATALASE* (6.94 logFC), important factors in the clearance of damaging ROS intermediates. Other dysregulated anti-oxidant genes were *ALDH1A3*, *ALDH3B1*, *AKR1C2*, *AKR1C1*, *CYP3A5*, *GSTT2*, and *GSTM1*. In contrast to the current hypothesis, our data suggest that this pro-oxidant signature may be an early event in the development of the cerebellum, rather than a progressive accumulation that affects the brain later in life, possibly coinciding with bursts of metabolic activity associated with the transition from a heavy reliance on glycolysis to oxidative phosphorylation. In further support of this, we find misregulation of a number of ERK1/2 substrates. This is of interest because ERK1/2 is critical in maintaining redox balance, and is a known target of ATM (Kim and Wong, 2009). We (Nayler et al., 2012) and others (Fu et al., 2008; Patel et al., 2011; Valentin-Vega et al., 2012) have previously demonstrated disruption at the level of the mitochondria in cells derived from A-T patients. It is worth noting also that ROS levels have been shown to negatively impact stem cell expansion in the bone marrow in an *Atm*-dependent fashion (Ito et al., 2004; D’Souza et al., 2012).

Another significantly altered network was *Synaptic vesicle cycle.* This is consistent with recent data indicating that *Atm*-deficiency causes disruption at the synapse, where ATM forms a complex with VAMP2 and SYNAPSIN-1 (Li et al., 2009; Siddoway et al., 2013). Li et al. (2009) showed that an *Atm*-deficient mouse model exhibits deficits in long-term potentiation and impaired rates of vesicular release hippocampal neurons. Subsequent work showed co-localization of ATM with presynaptic marker Piccolo, and impaired paired-pulse facilitation in *Atm*-deficient mice, indicative of presynaptic function deficit (Vail et al., 2016). Our transcriptome analysis reveals significant changes in the network constituents governing these processes occurring at the pre-synaptic nerve terminal, in particular *SYNAPSIN-1* and *SYNTAXIN-1B*, which encode two proteins critical in LTP (Hicks et al., 1997). It is unclear why A-T murine models do not recapitulate the full spectrum of neurological defects observed in the human condition. One possibility is that *Atm*-deficient mice succumb to lymphoid malignancy before neurodegenerative changes set in. Our data in a human model system support the synaptic vesicle defects identified in hippocampal neurons by Li et al. (2009) and suggest that this disruption in neurotransmission may also extends to cerebellar neurons, however, conformation will require additional functional studies.

An intriguing result from the differential expression pathway analysis was the perturbation of *Insulin Secretion* and *Diabetes type I* pathways. The implication of *Insulin secretion* (*ADCY2*, *ADCYAP1R1, ADCYAP1, ATP1A3, CAM2K2B, SNAP25, RAB3A, KCNJ1, PCLO, KCNMB2, ADCY8, GLP1R, GCK, ABCC8, SLC2A2*) and *Type I diabetes mellitus* (*GAD1, HLA-DRB1, HLA-DRB5, HLA-DQB1, HLA-C, HLA-DRA, HLA-A, HLA-DQA1, PTPRN2*) pathways are interesting given recent data implicating A-T as a metabolic syndrome (Aird et al., 2015). Chronic DNA damage has been linked with metabolic disorders in diseases of premature aging such as Gilford progeria and Werner syndrome (Shimizu et al., 2014). Furthermore, disrupted transcription-coupled repair has been shown to disrupt redox balance, nutrient sensing and energy metabolism, all of which have been implicated with ATM (Shaughnessy et al., 2014).

It remains to be seen to what extent the self-organizing capacity of cerebellar progenitors/differentiated progeny can recapitulate the cellular arrangement and synaptic connections of the cerebellum *in vivo*. Our limited electrophysiological data suggested that there were a number of different cell types present which either showed no activity, spontaneous activity or evoked activity only. It is possible that the non-responsive cells may be granule neurons, while firing cells may be Purkinje or other neuronal cell types. While our gene expression and morphological analysis are in support of the presence of these cell types, conclusive demonstration of this would require access to larger sample sizes and or methodologies such as Patch-Seq (Cadwell et al., 2016). Although differentiated neurons may represent a reduced form and functional network complexity compared to *in vivo* development (Yin et al., 2016), progress in the isolation of defined cerebellar neuronal and glial cell types and their controlled deposition into hydrogel based scaffolds (bioprinting) based on their *in vivo-*configuration and proportion may provide a potential solution. This will, however, require the identification of defined cell surface markers and/or genetic tagging of cerebellar developmental genes. An alternative approach pioneered by Muguruma et al. (2015) involves external stimulation of key developmental pathways through co-culture with mouse cerebellum which generates cerebellar structures with a substantial degree of complexity.

We believe our current data constitute a valuable resource for the development of protocols that will offer insight into improved differentiation and culture-conditions for expansion of iPSC-derived human cerebellar neurons, identification of stage specific biomarkers of human cerebellar development, and further understanding of cerebellar pathogenesis in A-T through meta-analyses with other datasets. Analysis of additional A-T iPSC lines, isogenic gene corrected A-T iPSC lines and single-cell transcriptome profiling of cerebellar neuronal cells will allow more accurate assessment of developmental maturity of individual cell types and further validation of disease-specific gene expression profiles in the future. Our study represents the first *in vitro* iPSC-derived human disease model for A-T and constitutes an important first step toward the generation of a therapeutically tractable cellular model for screening chemical compounds for treatment of A-T and perhaps other debilitating cerebellar ataxias.

## Author Contributions

SN and EW conceived the initial study. SN performed cell differentiation, immunohistochemistry, microscopy and staining, RNA purification, NGS library preparation, qPCR experiments and analysis, NGS data analysis and visualization. JP performed the statistical analysis with ABA datasets and designed the 3-d visualization method. DV designed early versions of the RNA-seq analysis pipeline with guidance from RT, performed NGS data analysis and visualization. ML contributed to data interpretation. OK provided data analysis and bioinformatics support and performed NGS data analysis and visualization with supervision from CW. RK performed and analyzed electrophysiology experiments. JS generated iPSC. SN and EW wrote the manuscript. All authors reviewed and edited the manuscript. All authors read and approved the final manuscript.

## Conflict of Interest Statement

The authors declare that the research was conducted in the absence of any commercial or financial relationships that could be construed as a potential conflict of interest. The reviewer JP and handling Editor declared their shared affiliation.

## Footnotes

1 http://www.stemformatics.org/Stemformatics_data_methods.pdf

2 http://www.stemformatics.org/datasets/view/6855

3 http://www.advaitabio.com/ipathwayguide

4 http://human.brain-map.org/static/download

5 https://plot.ly

6 www.stemformatics.org/Stemformatics_data_methods.pdf

7 https://plot.ly/∼PlotSam/9/

## Supplementary Material

**TABLE S1 |** Mapping statistics for RNA-seq samples.

**TABLE S2 |** Differential expression gene-list for AT30 day 34 vs. AT30 day 0.

**TABLE S3 |** Differential expression gene-list for C11 day 34 vs. AT30 day 0.

**TABLE S4 |** Differential expression gene-list for AT30 day 34 vs. C11 day 34.

**TABLE S5 |** gProfiler/g;Cocoa gene-ontology analysis for AT30 day 34 vs. AT30 day 0 and C11 day 34 vs. C11 day 0.

**TABLE S6 |** Statistical comparisons to Allen Brain Atlas datasets.
