# Supplementary material for: Human iPSC-Derived Cerebellar Neurons from a Patient with Ataxia-Telangiectasia Reveal Disrupted Gene Regulatory Networks
Source: Front Cell Neurosci. 2017 Oct 13;11:321. doi: 10.3389/fncel.2017.00321 (PMC5645492; doi:10.3389/fncel.2017.00321)
Supplement: Supplementary file 7 [file Data_Sheet_1.DOCX]

*Supplementary material*

**Human iPSC-derived Cerebellar Neurons From a Patient With ataxia-telangiectasia Reveal Disrupted Gene Regulatory Networks.**

**Sam P. Nayler^1^, Joseph E. Powell^3,8^, Darya P. Vanichkina^3^, Othmar Korn^1^, Christine A. Wells^1,7^, Refik Kanjhan^4^, Jian Sun^1^, Ryan J. Taft^3,5,6^, Martin F. Lavin^2^, Ernst J. Wolvetang*^1^**

***Correspondence:** Ernst Wolvetang: e.wolvetang@uq.edu.au

**Supplementary data**

**
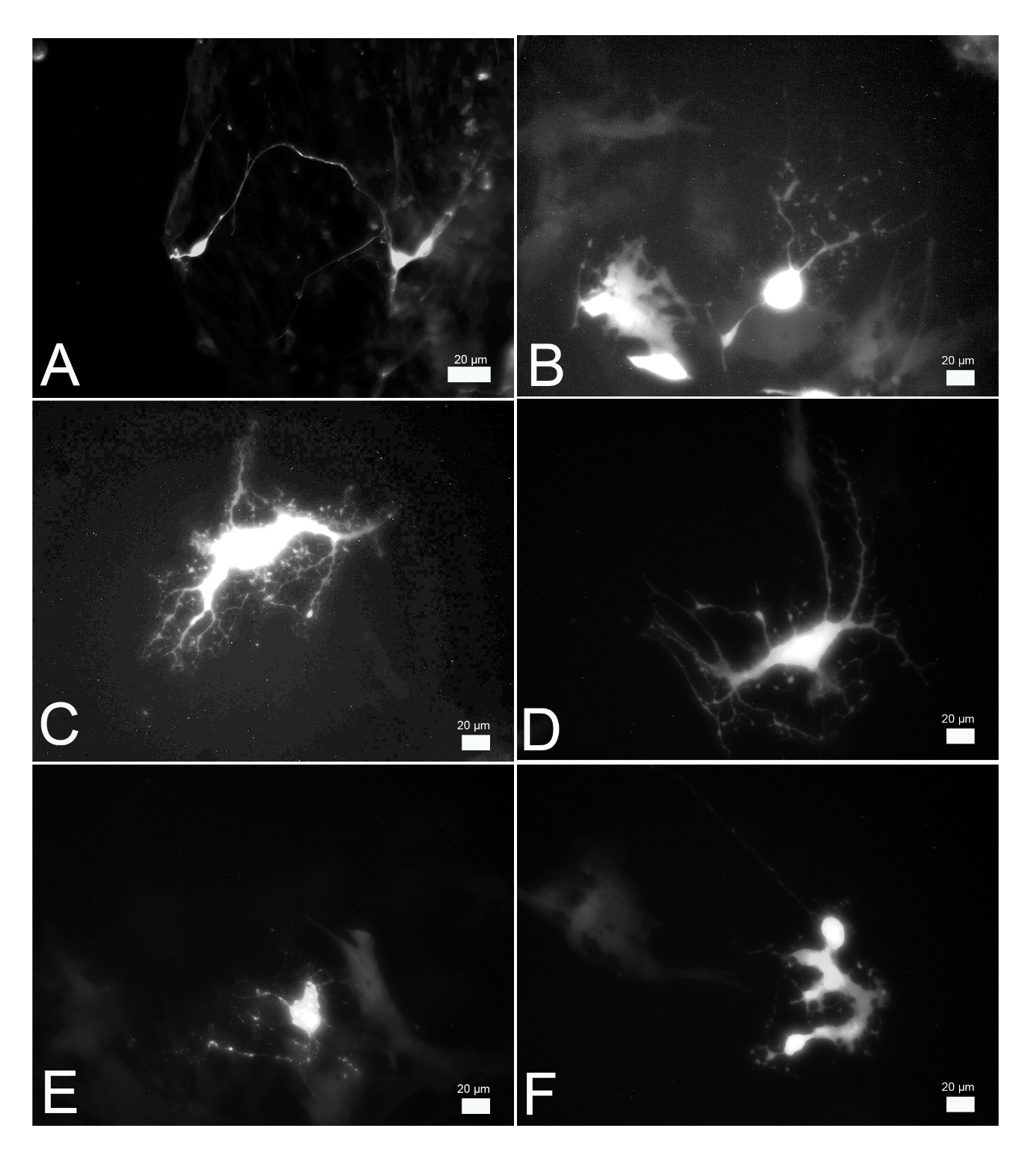
**

**Supplementary figure 1** – Fluorescently-labelled neurons following 34 days of differentiation. Labelled with pan-neuronal marker β-III-TUBULIN (A) or pmax-GFP (B-F) Scale bars are 20 µm.


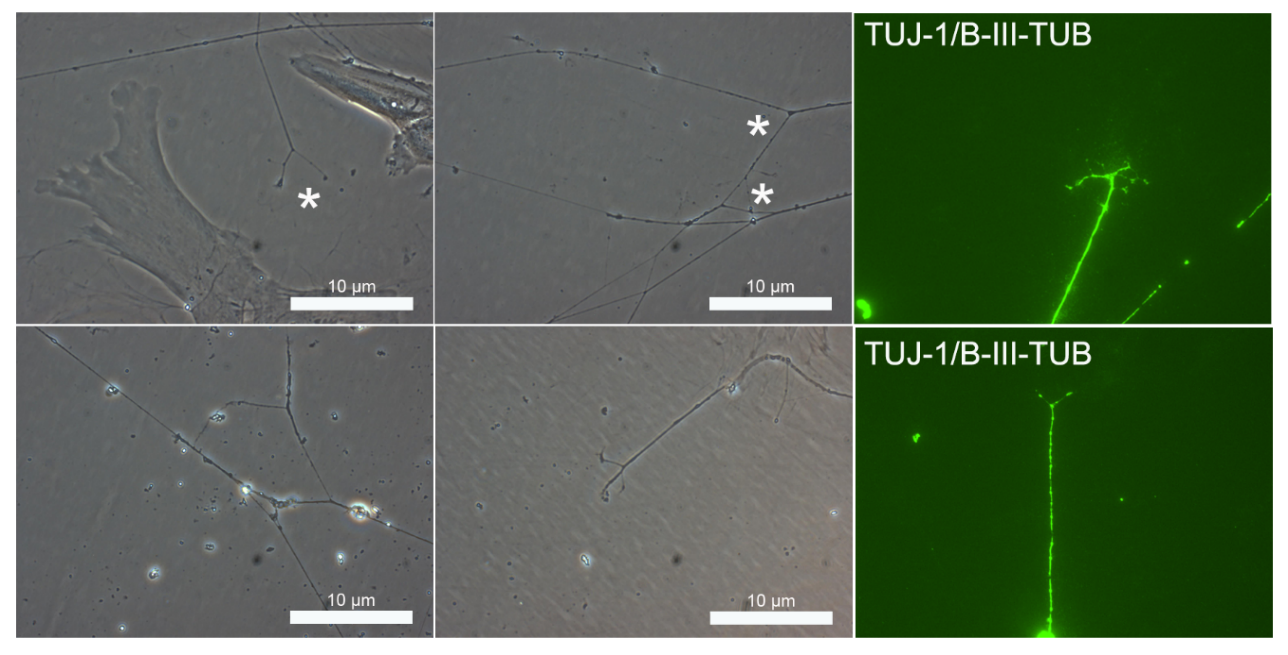


**Supplementary figure 2 –** Representative phase-contrast and immunofluorescent images showing T-shaped branching of neuronal axons. Scale bars are 10 µm.

| Identifier | Parental line | Genotype | Cell type |
| --- | --- | --- | --- |
| C11d0 | CRL2429 (C11) | wt | iPSC (d0) |
| C11d34 | CRL2429 (C11) | wt | Neurons (d34) |
| C32d0 | CRL 1502 (C32) | wt | iPSC (d0) |
| C32d34 | CRL 1502 (C32) | wt | Neurons (d34) |
| FBd0 | FB | wt | iPSC (d0) |
| FBd34 | FB | wt | Neurons (d34) |
| Hes3d0 | Hes3 | wt | iPSC (d0) |
| Hes3d34 | Hes3 | wt | Neurons (d34) |
| Hes3-Ed0 | Hes3-Envy | wt | iPSC (d0) |
| Hes3-Ed34 | Hes3-Envy | wt | Neurons (d34) |
| H9d0 | H9 | wt | iPSC (d0) |
| H9d34 | H9 | wt | Neurons (d34) |
| AT30d0 | AT30 | 8368delA & 7570delG | iPSC (d0) |
| AT30d34 | AT30 | 8368delA & 7570delG | Neurons (d34) |
| AT34d0 | AT34 | 7004delCA & 7886delTATTA | iPSC (d0) |
| AT34d34 | AT34 | 7004delCA & 7886delTATTA | Neurons (d34) |

**Supplementary figure 3 –** Details of iPSC lines used over the course of this study.


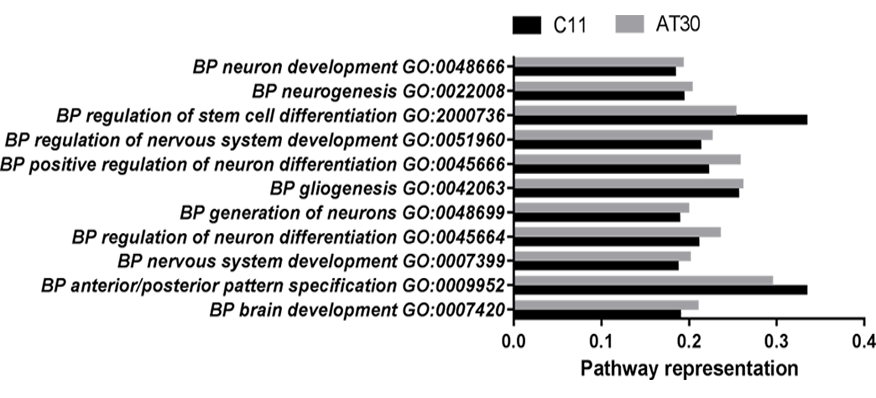


**Supplementary figure 4 –** Selected gene ontology results from g:Cocoa in C11 (black) and AT30 (grey) from respective D0vs34 differential expression profiles shows shared enrichment for processes including *Neuron development, neurogenesis, regulation of stem cell differentiation, regulation of nervous system development, positive regulation of neurondifferentiation, gliogenesis generation of neurons, regulation of neuron differentiation, nervous system development, anterio/posterior pattern specification & brain development*. Pathway representation (ie number of differentially expressed genes/all genes in pathway) is shown.


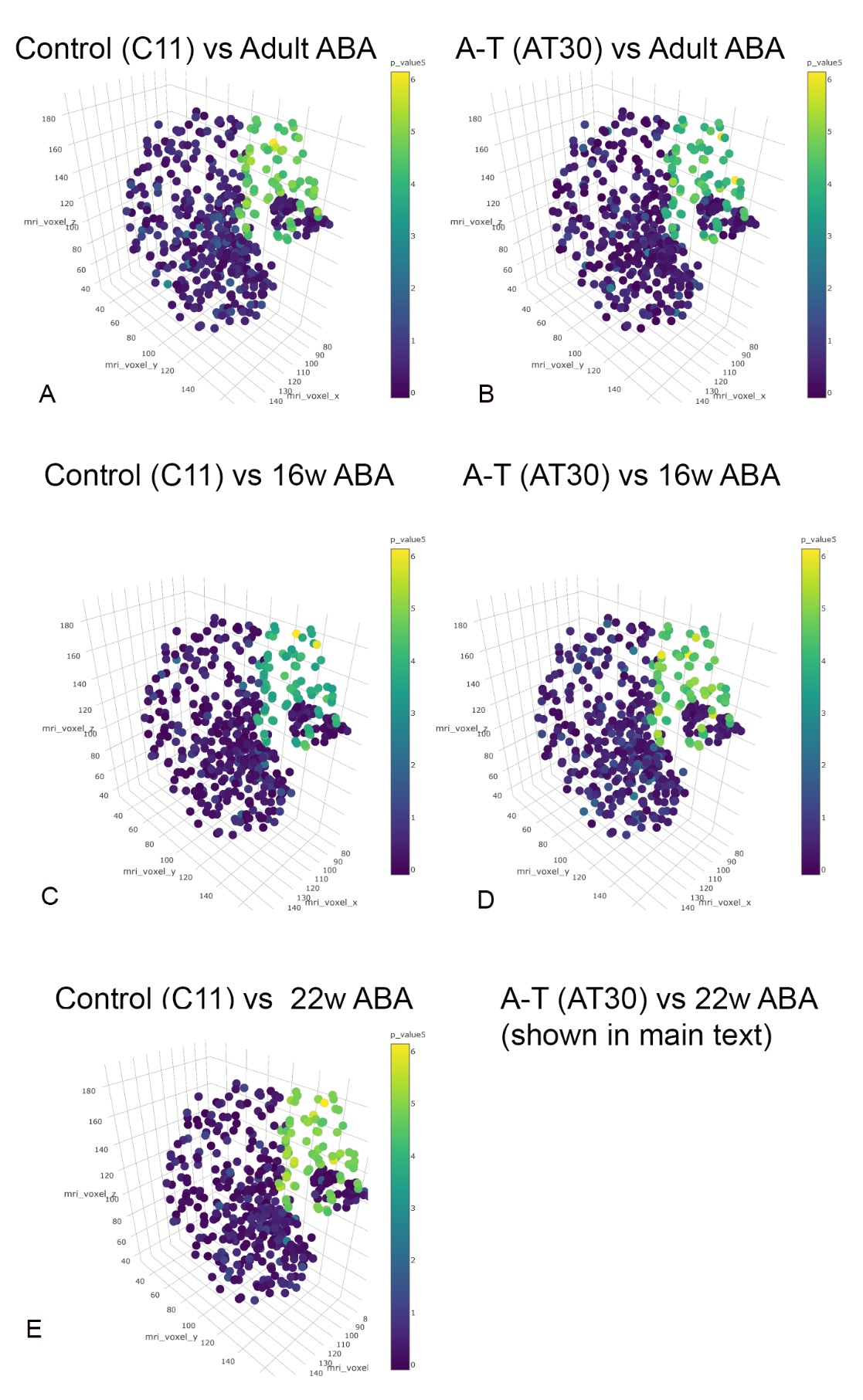


**Supplementary figure 5 –** 3D reconstructions of Allen Brain Atlas brain shows –log *p*-values plotted against voxel coordinates for relevant microdissected region. A) C11 vs Adult ABA B) AT30 vs Adult ABA C) C11 vs 16 week post-conception D) AT30 vs 16 week post-conception. E) C11 vs 22 week post-conception. Legend shows –log *p*-values.


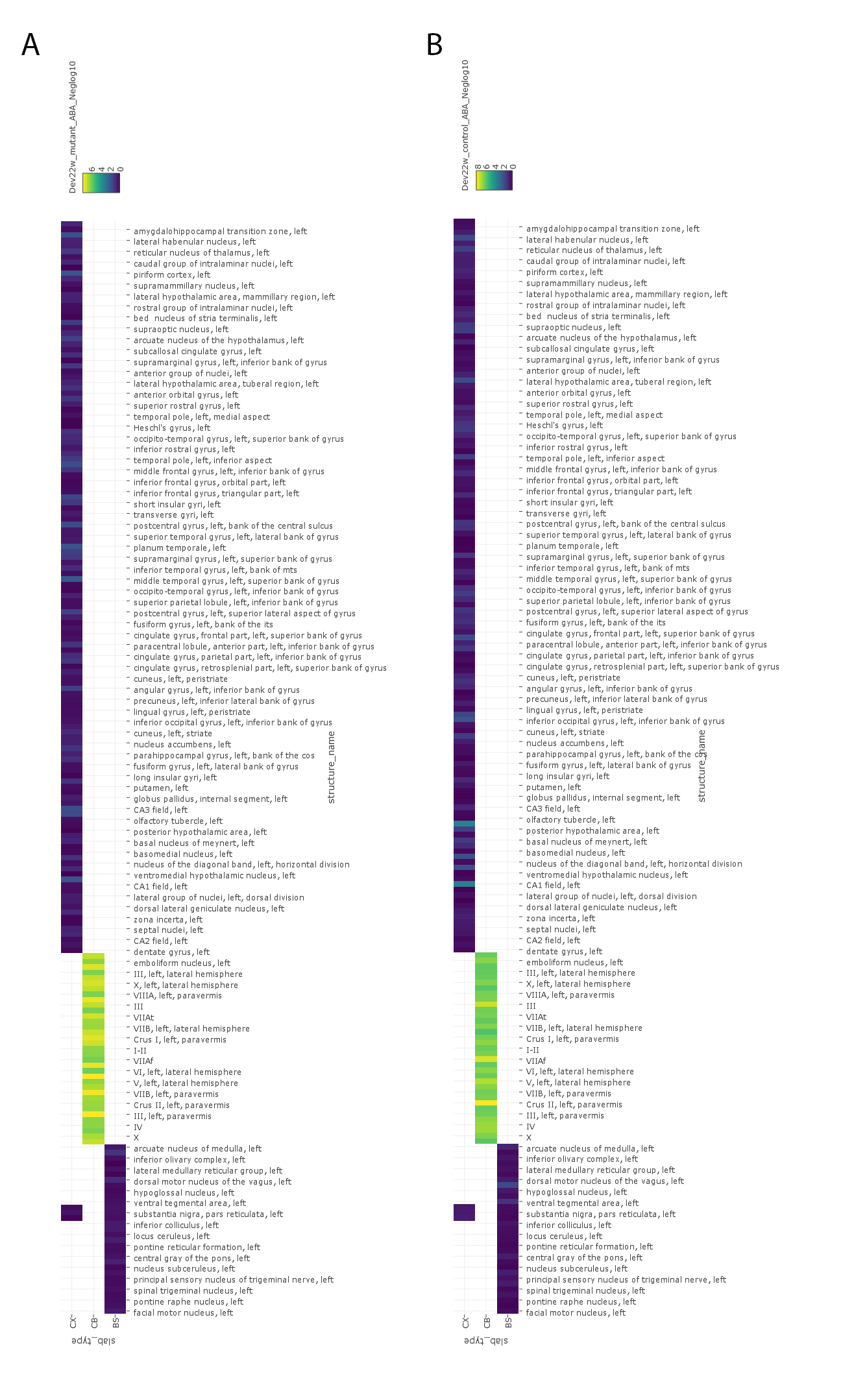


**Supplementary figure 6 –** Comparison between ABA 22 pcw data and A) AT30 & B) C11 RNA Seq data. Heatmap shows –log *p*-values for gross (y-axis) and fine brain regions (x-axis). Legend shows colour scale for –log *p*-values.


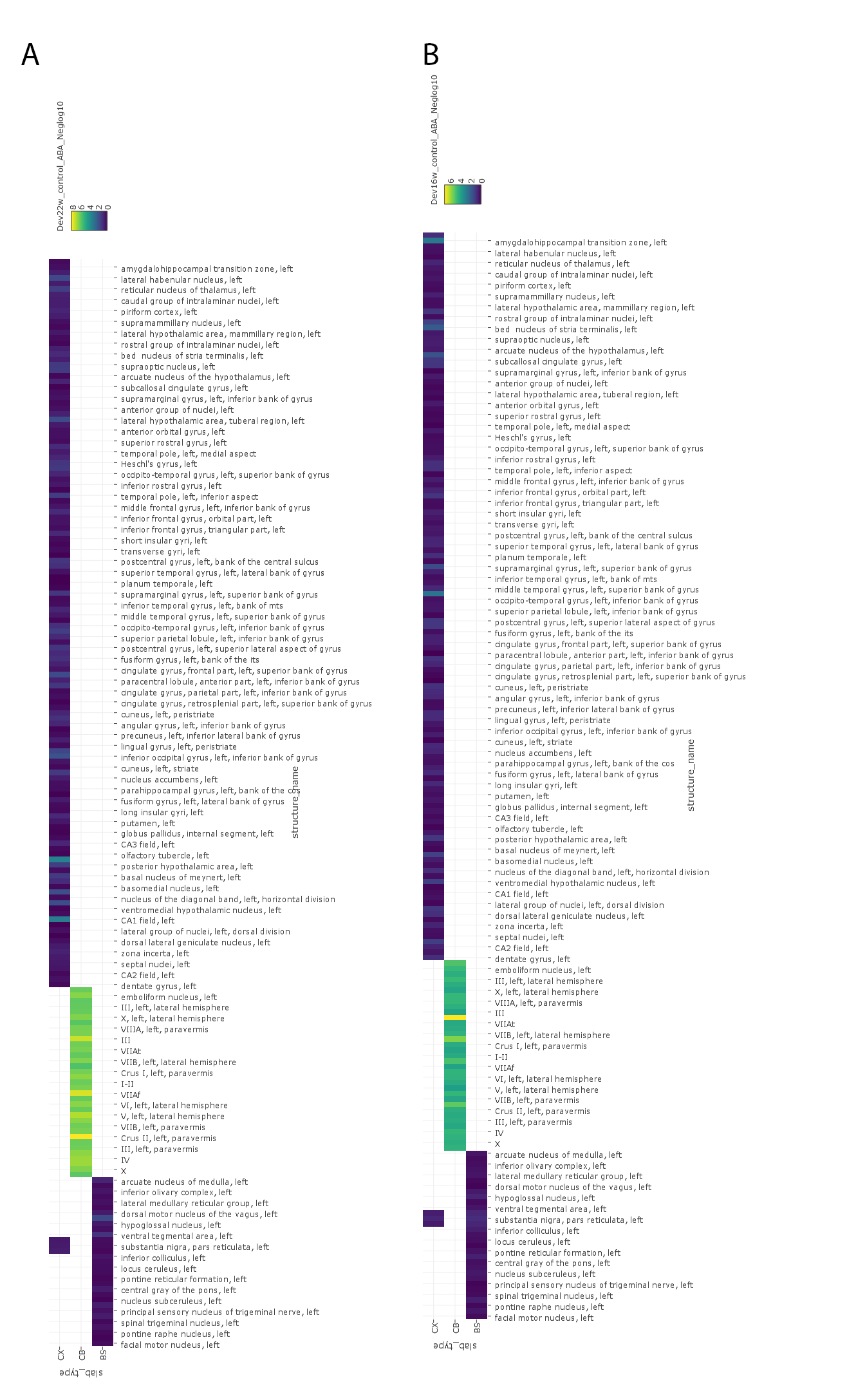


**Supplementary figure 7 -** Comparison between ABA 16 pcw data and A) AT30 & B) C11 RNA Seq data. Heatmap shows –log *p*-values for gross (y-axis) and fine brain regions (x-axis). Legend shows colour scale for –log *p*-values.


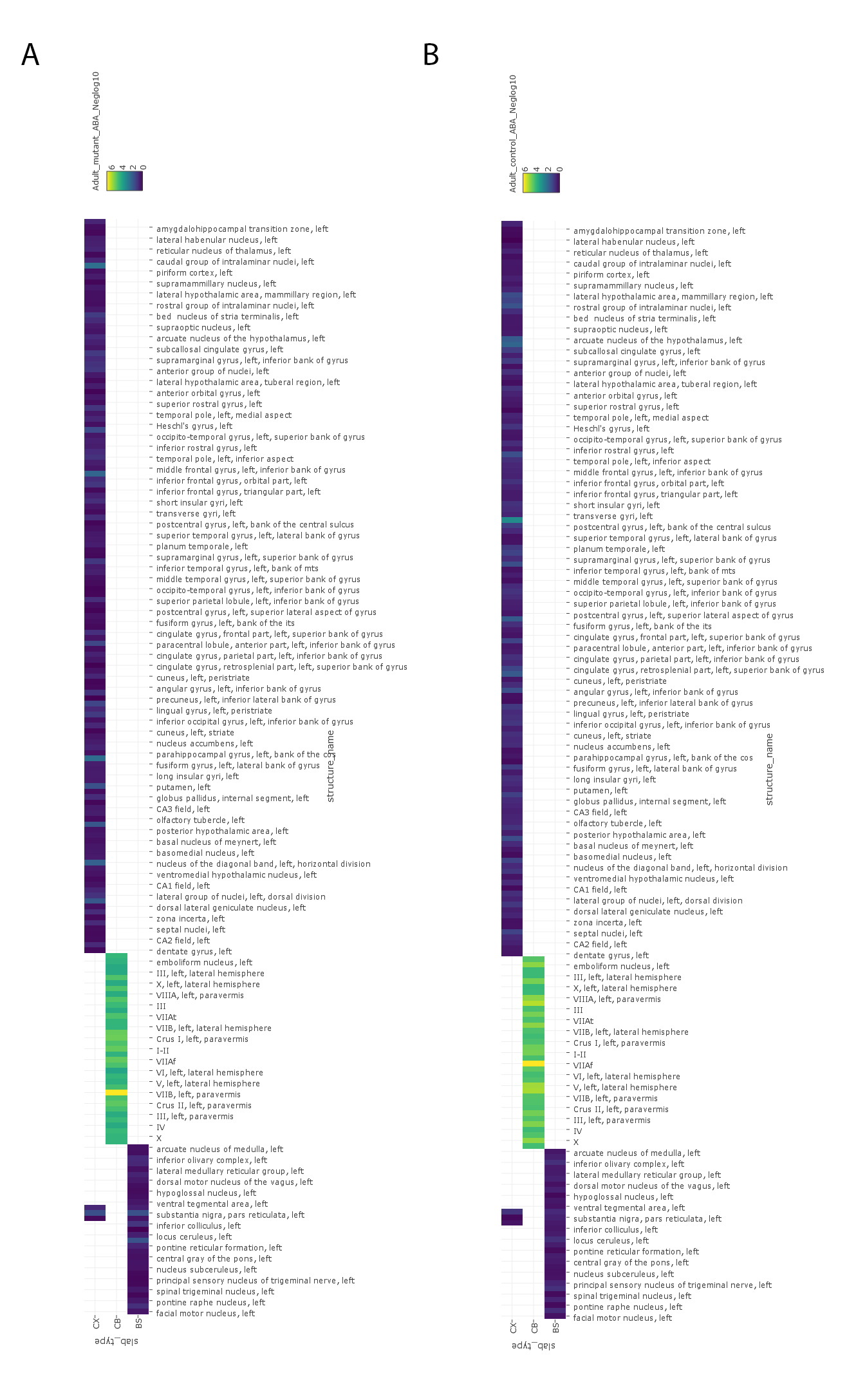


**Supplementary figure 8 -** Comparison between ABA Adult data and A) AT30 & B) C11 RNA Seq. data. Heatmap shows –log *p*-values for gross (y-axis) and fine brain regions (x-axis). Legend shows colour scale for –log *p*-values.


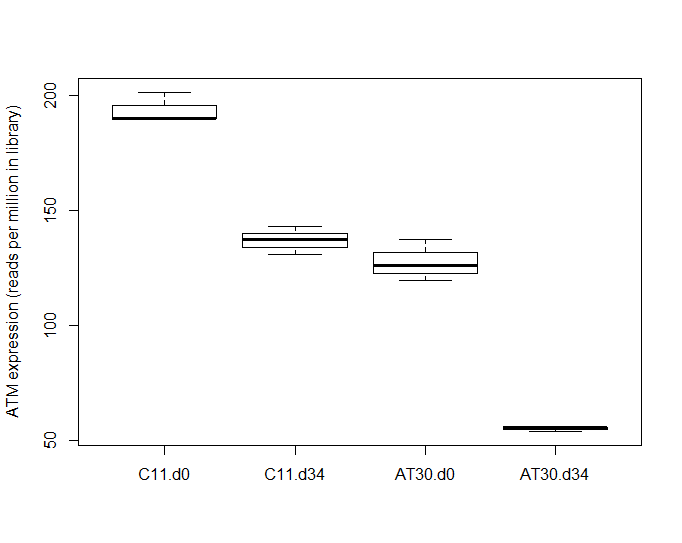


**Supplementary figure 9 –** Library size-adjusted expression of *ATM* across differentiation time course in control (C11) and A-T (AT30) cells.


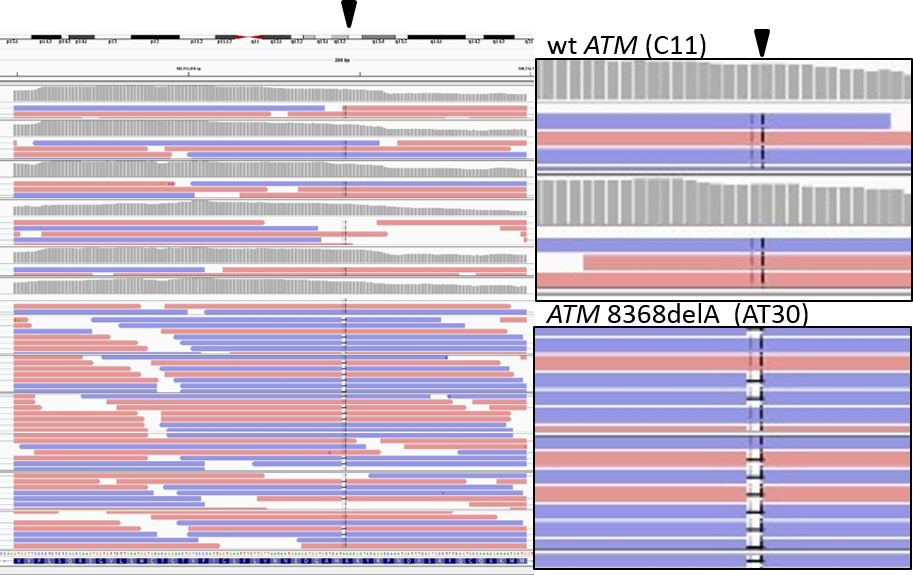


**Supplementary Figure 10 -** IGV snapshot demonstrating the presence of *ATM* 8368delA mutation in AT30 (lower half of diagram) but absent in C11 controls (upper half of diagram).


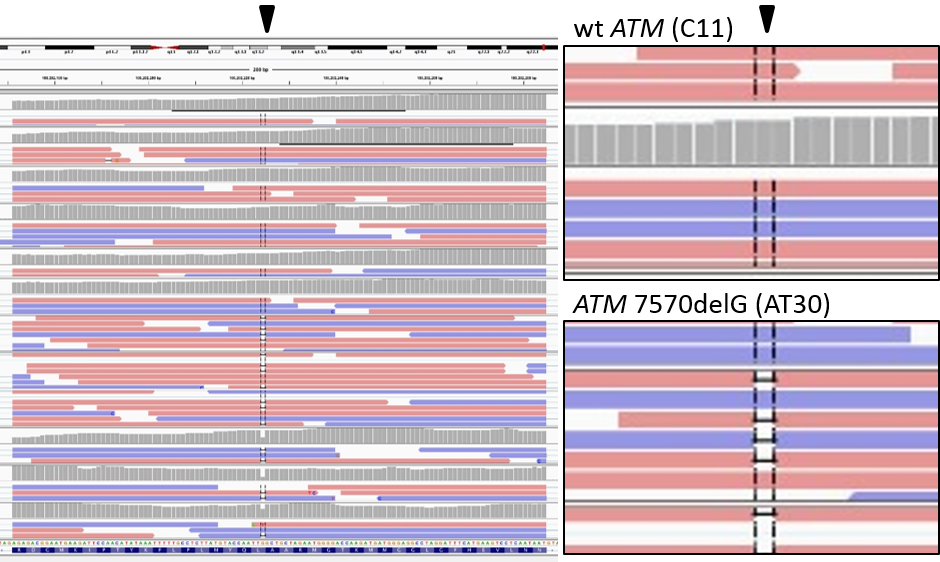


**Supplementary figure 11 -** IGV snapshot demonstrating the presence of *ATM* 7570delG mutation in AT30 (lower half of diagram) but absent in C11 controls (upper half of diagram).


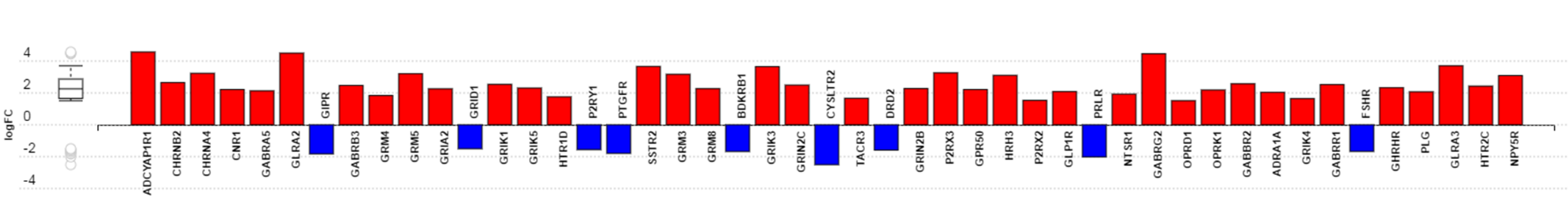


**Supplementary figure 12 -** Differentially-expressed genes implicated in *Synaptic vesicle cycle* pathway (4.85E-04). Y-axis shows perturbation values in AT30 compared to C11.


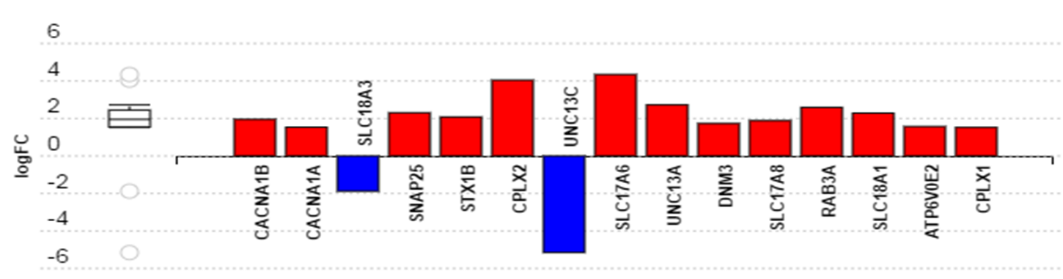


**Supplementary figure 13 -** *Neuroactive ligand-receptor interaction* pathway (3.66E-04). Y-axis shows perturbation values in AT30 compared to C11.


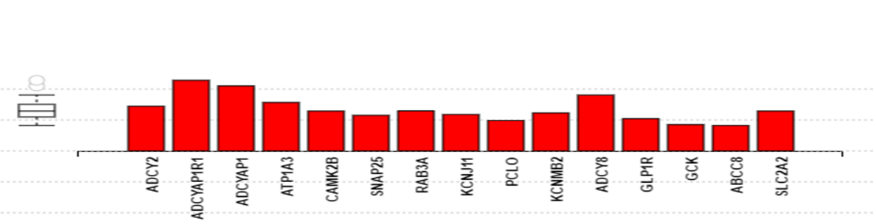


**Supplementary figure 14 -** *Insulin secretion* pathway (0.001, 4E). Y-axis shows perturbation values in AT30 compared to C11.
